# Cross-sectional study of women’s representation in leadership positions in Turkish medical schools, academic departments, specialty boards, conferences and journals in 2023

**DOI:** 10.1136/bmjopen-2024-089298

**Published:** 2025-10-13

**Authors:** Fatma Sıla Eroğlu, Sevil Buket Koyuncu, Beyza Erkan, Yavuz Selim Kıyak

**Affiliations:** 1 Gazi University Faculty of Medicine, Ankara, Turkey; 2 Medical Education and Informatics, Gazi University Faculty of Medicine, Ankara, Turkey

**Keywords:** Education, Medical, Schools, Medical, Health Education, Health policy, Organisation and Administration

## Abstract

**Abstract:**

**Objectives:**

Ensuring gender equity in leadership is crucial for fair representation and diversity in academic medicine. This study aims to investigate the representation of women in leadership positions in Turkish academic medicine, including medical schools, specialty boards, conferences and medical journals.

**Design and setting:**

A cross-sectional study was conducted between August and December 2023. The study analysed data from members of medical faculties, specialty boards, medical conferences and medical journals across Turkey. The source of information was publicly accessible websites.

**Participants:**

The study included data from 17 939 members of 113 medical faculties, 112 specialty boards, 73 medical conferences and 246 medical journals in Turkey.

**Interventions:**

This study has no interventions.

**Results:**

Women made up 40.4% of all medical-school faculty but only 22.5% of deans (95% CI 15.5 to 31.6; p<0.001) and 13.6% of surgical-division chairs (7.8–22.7; p<0.001). Vice-deanships (38.5%; 31.4–45.9) did not differ from baseline (p=0.62). Among 49 departments, significant shortfalls were confined to 6, notably paediatrics (36.1% chairs vs 57.9% faculty, p<0.001) and gynaecology-obstetrics (23.2% vs 41.6%, p=0.004). On specialty boards, women held 30.5% of president/chair posts versus 36.7% of all board seats (p=0.18); comparable figures for conferences were 34.2% versus 41.4% (p=0.20). Women occupied 27.2% of editor-in-chief positions, significantly below their 32.9% share of other editorial roles (95% CI 22.8 to 32.0; p=0.007).

**Conclusions:**

Turkey’s academic medicine pipeline contains substantial numbers of women, yet marked gaps persist in senior positions. Bridging these gaps will require targeted policies that look beyond overall workforce proportions to the specific decision-making roles where shortfalls remain.

**STRENGTHS AND LIMITATIONS OF THIS STUDY**

- Diverse data sources like faculty, board, conference and journal websites provide a broad dataset.
- Cross-sectional design cannot establish causality.
- Potential inaccuracies due to missing or outdated information in publicly available data sources.
- Risk of misgendering individuals based on available photos and internet searches.

## Introduction

 Gender equity is a fundamental human right, emphasising the principle that every individual, regardless of their gender, deserves equal chances, treatment and resources. The pursuit of gender equity, as outlined in the United Nations’ fifth Sustainable Development Goal, emphasises the need for equal representation of women in leadership roles across various sectors. As highlighted in the United Nations Sustainable Development Goals Report 2023, despite progress, women globally held 28.2% of management positions in 2021, with over 140 years projected to achieve gender parity in these positions.^1^

This concern applies within the specific context of Turkey as well. According to The Global Gender Gap Index 2023, there are 19.6% of women in leadership positions such as legislators, senior officials and managers in the country.^2^ Turkey’s Sustainable Development Goals second National Review Report further underscores the urgency for understanding and improving women’s representation, with statistics indicating a 2019 employment rate of 28.3% for women, a workforce participation rate of 34% and a mere 16.7% representation in leadership positions.^3^ As a response to this issue, the Turkish government officially aims to enhance women’s representation in employment, workforce participation and leadership roles as part of its 11th Development Plan.^4^

Given that leadership positions in academic medicine are varied, there exists a significant research gap in comprehensively addressing women’s representation in these positions, not only in the Turkish context but also on a global scale. Over 90% of studies on women’s leadership in academic medicine focus on the Global North,^5^ predominantly providing data on Western countries, while perspectives from the Global South remain under-represented in academic medicine research.^6^ Therefore, this paper aims to highlight the crucial issue of women’s representation in leadership positions within Turkish academic medicine. The research question of this study is as follows: to what extent are women represented in formal leadership positions in Turkish (1) medical schools, (2) medical specialty boards and conferences and (3) medical journals?

Through a thorough examination of existing public sources, our objective is to make a meaningful contribution to the existing literature on gender equity by providing findings from a Global South country. This study aims to provide a comprehensive analysis of the current state of academic medicine towards a future where leadership positions are not confined by gender boundaries. Ultimately, it will not only benefit the medical community in Turkey but will also give valuable insights to a global audience.

## Methods

This is a cross-sectional study. We used the STROBE (Strengthening the Reporting of Observational Studies in Epidemiology) cross-sectional reporting guidelines.^7^ The data collection process was carried out between August and December 2023. To analyse the representation of women in leadership and management positions in academic medicine in Turkey, our research focused on three primary areas: medical faculties, professional societies (specialty boards) and conferences and medical journals.

### Data collection tools

#### Medical faculties

Initially, we identified all medical faculties in Turkey, including both private and public universities, based on the ‘Council of Higher Education’ (YÖK) registry in Turkey.^8^ We found a total of 113 medical faculties, of which 15 were excluded during the research due to the unavailability of a website or necessary information.

Following that, we created an Excel file and added 113 medical schools for data collection. In order to record the number of individuals and the number of women in various positions, we inserted columns into the Excel file for Dean, Vice Dean, Faculty Board Member, Faculty Executive Committee Member, Chair of Basic Sciences Division, Chair of Internal Sciences Division and Chair of Surgical Sciences Division positions. We also inserted three columns into the Excel file for each 1 of the 49 departments: gender of the department chair, number of faculty members and number of women faculty members. All data collected on the medical faculties was recorded in this Excel file.

#### Professional societies (specialty boards) and conferences

The selection of professional societies for the study was based on the list available on the Turkish Medical Association website as of 1 August 2023, with a total of 112 societies (specialty boards). We created another Excel file and added the names of these societies to the file for data collection. By examining the websites of these professional societies, we gathered data on the number of individuals holding the positions and the number of women in these positions. The positions were Chair/President (including co-chair), Vice Chair/President, General Secretary, Treasurer and Executive Committee Member. We inserted columns into the Excel file for these positions. After identifying the board positions on the websites, the latest conference organised by each society was determined. This encompassed the Conference Chair (including co-chair), Vice Chair, General Secretary and Conference Committee Member. We inserted columns into the Excel file for 112 societies. All data regarding the societies and conferences was recorded in this Excel file.

#### Medical journals

To identify medical journals in Turkey, we conducted a search using Web of Science and Turkish index (TRDizin), a national database, to compare journals at national and international levels. The search was conducted on 31 July 2023. In Web of Science, we applied a ‘Turkey’ filter and found 306 journals, each journal was examined and 96 were included in the study because they were medical journals.

In the TR (Turkish) Index, we applied filters for ‘science’ and ‘medicine’, identifying 257 journals. We excluded non-medical journals and those related to other health sciences, leaving 217 journals for the study. After comparing both search lists, eliminating non-medical journals and duplicates, a total of 246 medical journals were included in another Excel file for data collection.

We examined the editor/editor-in-chief, associate/deputy/co-/managing editor, section editor and editorial board member positions in each journal to determine the number of positions and the number of women in each role. The count of women and total numbers were charted in the Excel file.

### Data collection process

The data collection process involved four researchers. In the pilot phase, to develop a shared understanding between the researchers, each researcher was assigned to a specific medical faculty, a professional society and its conference and a medical journal. Following an independent data collection process on women’s representation in these entities, the researchers compared and discussed their findings. During this pilot phase, researchers collaborated to assess the feasibility of the data collection process and resolve any conflicts. Throughout this process, researchers were responsible for recording positions and the number of women in the Excel files mentioned above. The Excel files were updated considering the insights during this process. Any conflicts were discussed collectively, and decisions were made as a group. It was not difficult to establish a shared understanding because this current research constitutes the second phase of a research project in which a scoping review^9^ on leadership and management positions in academic medicine was conducted together with this team as the first phase.

Following the establishment of a shared understanding and the final version of data collection tools, data were collected through the official websites and gender recognition was based on information available on websites, photos or internet searches (inference from typical Turkish given names and visual inspection of photographs). If a relevant position was not listed or the leader’s gender was absent, we applied a supplementary strategy: searching Turkish-language internet pages, university bulletins and public posts. Blank cells were used as the indicator of missing data.

### Statistical analysis

We faced two tiers of missing data across all four data sets (medical schools, specialty boards, conferences, journals). First, a small group of institutions (eg, 15 out of 128 medical schools) had no accessible data even after our efforts to find, so they were excluded list-wise. Second, information that could not be located on an institution’s public website was coded as missing (blank cell) at the time of extraction. Across the entire data set this affected ≤12% of medical-school leadership cells, ≤ 5% of specialty-board and conference cells and <3% of journal-editorial cells. All denominators in the results tables therefore reflect the number of non-missing observations, and 95% CIs were calculated to account for the reduced sample size where relevant.

For medical school data, percentages were calculated for administrative positions (dean, vice dean, faculty board member, faculty executive committee member, division hair) in 49 departments (faculty member, department chair). We used Pearson’s χ² test with continuity correction to compare the proportion of women in each leadership position to the baseline faculty distribution. For each of the positions (including 49 departments), we built a 2×2 contingency table of gender (women vs men) by role (target position vs all other academic staff in the same department). The primary comparison when conducting Pearson’s χ² test with continuity correction was the proportion of women in the chair post versus their proportion among all faculty in that department.

Regarding data obtained from specialty boards and conferences, percentages of women’s representation were calculated for administrative positions in boards and conferences (chair/president, vice chair/president, general secretary, treasurer, executive committee member, conference committee member). We used Pearson’s χ² test with continuity correction each time comparing the gender distribution in the position to that in all other positions on the same board or conference.

By following the classification widely used in Turkey, we used ‘Internal Sciences’ to refer to clinical, non-surgical disciplines, typically encompassing specialties such as internal medicine, paediatrics, neurology and similar fields. ‘Surgical Sciences’ refers to any specialty that involves surgical procedures, such as general surgery, ophthalmology or related fields like pathology that support surgical work. ‘Basic Sciences’ refers to non-clinical specialties, which include disciplines like anatomy, physiology, biochemistry and others that do not directly involve patient care.

Finally, from the journal data, percentages were calculated for different positions (editor-in-chief/editor, associate/deputy/co-/managing/section editors, editorial board member). For the editor-in-chief position, we constructed a 2×2 contingency table of gender (women vs men) by role (the position vs all other editorial positions pooled across the same 246 journals). We used Pearson’s χ² test with continuity correction.

Jamovi (V.2.2.5), an R-based open-source software,^10^ was used for the statistical analysis. A p value threshold of 0.05 was used to determine statistical significance.

### Patient and public involvement

Patients and the public were not involved at any stage of this study.

## Results

### Medical schools

Of the 128 medical schools identified nationwide, we were able to obtain data for 113 (88%). In Turkish medical schools, women accounted for 22.5% of deans and 38.5% of vice deans. Table 1 presents the proportion of women in each leadership role compared with their overall representation among faculty (40.4%), highlighting statistically significant under-representation in most top administrative positions.

**Table 1:** Women’s representation in the top administrative positions in Turkish medical schools, compared with overall faculty women proportion

Administrative positions (n=medical schools)	Total number of positions	Number of women (%)	95% CIs (%)	P value*
Dean (n=102)	102	23 (22.5)	15.5 to 31.6	<0.001
Vice Dean (n=97)	182	70 (38.5)	31.4 to 45.9	0.62
Faculty Board Member (n=96)	919	318 (34.6)	31.6 to 37.7	<0.001
Faculty Executive Committee Member (n=97)	685	239 (34.9)	31.4 to 38.5	0.003
Chair of Basic Sciences Division (n=85)	85	25 (29.4)	20.8 to 39.8	0.048
Chair of Internal Sciences Division (n=83)	83	19 (22.9)	15.2 to 32.6	0.002
Chair of Surgical Sciences Division (n=81)	81	11 (13.6)	7.8 to 22.7	<0.001

* Pearson’s χ² test with continuity correction.

Women accounted for 40.4% of all faculty members (max: 72.0% in dermatology, min: 0.6% in urology). Women’s representation in department chair positions was 34.6% (max: 63.8% in dermatology, min: 0% in urology and orthopaedics). Table 2 presents the findings according to departments.

**Table 2:** Women’s representation in Turkish medical schools according to departments

Departments (n=medical schools)	Number of faculty members	Number of women (%)	Faculty members 95% CIs (%) (low - high)	Number of chair positions	Number of women chairs (%)	Chair positions 95% CIs (%) (low - high)	*P value
**Internal sciences**							
Aerospace Medicine (n=1)	1	0 (0)	0 to 79.3	1	0 (0)	0 to 79.3	N/A
Cardiology (n=93)	692	115 (16.6)	14 to 19.6	68	7 (10.3)	5.1 to 19.8	0.237
Child and Adolescent Psychiatry (n=67)	171	116 (67.8)	60.5 to 74.4	54	34 (63.0)	49.6 to 74.6	0.619
Dermatology (n=87)	293	211 (72.0)	66.4 to 76.6	69	44 (63.8)	52 to 74.1	0.245
Emergency Medicine (n=86)	360	90 (25.0)	20.8 to 29.7	66	15 (22.7)	14.3 to 34.2	0.811
Family Medicine (n=70)	192	100 (52.1)	45 to 59	58	30 (51.7)	39.2 to 64.1	1.000
Forensic Medicine (n=70)	152	36 (23.7)	17.6 to 31	57	12 (21.1)	12.5 to 33.3	0.827
Infectious Diseases and Clinical Microbiology (n=83)	332	193 (58.1)	52.8 to 63.3	66	31 (47.0)	35.4 to 58.8	0.125
Internal Medicine (n=95)	1549	571 (36.9)	34.5 to 39.3	69	17 (24.6)	16 to 36	0.053
Medical Ecology and Hydroclimatology (n=5)	8	4 (50.0)	21.5 to 78.5	5	3 (60.0)	23.1 to 88.2	1.000
Neurology (n=89)	497	273 (54.9)	50.5 to 59.2	65	34 (52.3)	40.4 to 64	0.790
Nuclear Medicine (n=63)	180	81 (45.0)	37.9 to 52.3	51	19 (37.3)	25.3 to 51	0.409
Paediatrics (n=94)	1464	848 (57.9)	55.4 to 60.4	72	26 (36.1)	26 to 47.6	<0.001
Physical Medicine and Rehabilitation (n=86)	411	253 (61.6)	56.8 to 66.1	46	24 (52.2)	54 to 75.8	0.596
Psychiatry (n=87)	392	187 (47.7)	42.8 to 52.6	66	28 (42.4)	49.6 to 74.6	0.619
Public Health (n=86)	262	140 (53.4)	47.4 to 59.4	67	34 (50.9)	39.1 to 62.3	0.798
Pulmonology (n=82)	404	236 (58.4)	53.6 to 63.1	66	28 (42.4)	31.2 to 54.4	0.022
Radiation Oncology (n=55)	194	99 (51.0)	44 to 58	47	25 (53.2)	39.2 to 66.7	0.918
Radiology (n=91)	687	266 (38.7)	35.1 to 42.4	67	20 (29.9)	20.2 to 41.7	0.195
Sports Medicine (n=17)	40	5 (12.5)	5.5 to 26.1	16	1 (6.3)	1.1 to 28.3	0.838
Undersea and Hyperbaric Medicine (n=5)	10	3 (30.0)	10.8 to 60.3	5	1 (20.0)	3.6 to 62.4	1.000
**Basic sciences**							
Anatomy (n=95)	396	162 (40.9)	38.9 to 49	73	19 (26.0)	17.3 to 37.1	0.007
Biochemistry (n=93)	434	239 (55.1)	50.4 to 59.7	71	28 (39.4)	28.9 to 51.1	0.020
Biophysics (n=80)	175	77 (44.0)	36.9 to 51.4	62	26 (41.9)	30.5 to 54.3	0.894
Biostatistics (and Medical Informatics) (n=77)	169	75 (44.4)	37.1 to 51.9	62	26 (41.9)	30.5 to 54.3	0.856
Histology and Embryology (n=97)	343	234 (68.2)	63.1 to 72.9	75	46 (61.3)	50 to 71.5	0.311
Immunology (n=17)	37	19 (51.4)	35.9 to 66.6	14	7 (50.0)	26.8 to 73.2	1.000
Medical Biology (n=90)	359	227 (63.2)	58.1 to 68.1	67	34 (50.9)	39.1 to 62.3	0.074
Medical Education (and Informatics) (n=61)	181	91 (50.3)	43.1 to 57.5	54	34 (63.0)	49.6 to 74.6	0.138
Medical Genetics (n=71)	187	97 (51.9)	44.7 to 58.9	58	24 (41.4)	29.6 to 54.2	0.213
Medical History and Ethics (n=62)	97	50 (51.6)	41.7 to 61.2	50	22 (44.0)	31.2 to 57.7	0.488
Medical Microbiology (n=94)	429	237 (55.2)	50.5 to 59.9	72	33 (45.8)	34.8 to 57.3	0.176
Medical Pharmacology (n=89)	294	137 (46.6)	41 to 52.3	69	27 (39.1)	28.5 to 50.9	0.323
Parasitology (n=19)	55	23 (41.8)	29.7 to 55	17	8 (47.1)	26.2 to 69	0.919
Physiology (n=95)	384	194 (50.5)	45.5 to 55.5	71	29 (40.9)	30.2 to 52.5	0.171
**Surgical sciences**							
Anaesthesiology and Reanimation (n=91)	745	387 (52.0)	48.4 to 55.5	68	29 (42.6)	31.6 to 54.5	0.180
Cardiovascular Surgery (n=89)	387	30 (7.9)	5.5 to 10.9	69	4 (5.8)	2.3 to 14	0.748
General Surgery (n=95)	759	60 (7.9)	6.2 to 10	72	1 (1.4)	0.2 to 7.5	0.073
Gynaecology and Obstetrics (n=94)	682	284 (41.6)	38 to 45.4	69	16 (23.2)	14.8 to 34.4	0.004
Maxillofacial Surgery (n=3)	5	1 (20.0)	3.6 to 62.4	3	1 (33.3)	6.1 to 79.2	1.000
Medical Pathology (n=90)	479	341 (71.2)	67 to 75.1	72	39 (54.2)	42.7 to 65.2	0.006
Neurosurgery (n=86)	404	22 (5.5)	3.6 to 8.1	63	2 (3.2)	0.9 to 10.9	0.651
Ophthalmology (n=91)	543	221 (40.7)	36.6 to 44.9	67	26 (38.8)	28 to 50.8	0.868
Orthopaedics (n=91)	560	8 (1.4)	0.7 to 2.8	69	0 (0)	0 to 5.3	0.667
Otolaryngology (n=88)	461	105 (22.8)	19.2 to 26.8	66	10 (15.2)	8.4 to 25.7	0.214
Paediatric Surgery (n=78)	226	46 (20.4)	15.6 to 26.1	62	13 (21.0)	12.7 to 32.6	1.000
Plastic Surgery (n=71)	189	33 (17.5)	12.7 to 23.5	56	13 (23.2)	14.1 to 35.8	0.439
Thoracic Surgery (n=74)	194	26 (13.4)	9.3 to 18.9	63	6 (9.5)	4.4 to 19.3	0.555
Urology (n=93)	474	3 (0.6)	0.2 to 1.8	68	0 (0)	0 to 5.3	0.790
Total	17 939	7256 (40.44)		2763	956 (34.60)		

* Pearson’s χ² test with continuity correction.

Pearson χ² testing showed that, out of the 49 clinical and basic-science departments, women were significantly under-represented in the chair position in six departments. The most pronounced gaps were seen in paediatrics (36.1% female chairs vs 57.9% female faculty, p<0.001), gynaecology and obstetrics (23.2% female chairs vs 41.6% female faculty, p=0.004), medical pathology (54.2% female chairs vs 71.2% female faculty, p=0.006) and anatomy (26.0% female chairs vs 40.9% female faculty, p=0.007). No department showed a statistically significant excess of women in chair roles, and in the remaining 43 departments the gender distribution of chairs mirrored their departmental faculty baseline.

### Boards and conferences

Women were not significantly under-represented as chairs in either boards (p=0.18) or conferences (p=0.20) relative to their overall share of board membership or conference committee membership. Table 3 presents women’s representation on specialty boards and conference positions.

**Table 3:** Women representation in administrative positions in Turkish medical specialty boards and medical conferences organised by the boards

	Total number of positions	Number of women (%)	95% CIs
Positions (n=boards)			
Chair/President (including co-chair) (n=104)	105	32 (30.5)	22.5 to 39.8
Vice Chair/President (n=76)	80	28 (35.0)	25.5 to 45.9
General Secretary (n=98)	98	44 (45.0)	35.4 to 54.8
Treasurer (n=92)	92	32 (34.8)	25.8 to 44.9
Executive Committee Member (n=103)	842	309 (36.7)	25.8 to 44.9
Positions (n=conferences)			
Chair (including co-chair) (n=70)	76	26 (34.2)	24.5 to 45.4
Vice Chair (n=21)	22	6 (27.3)	13.2 to 48.2
General Secretary (n=56)	79	39 (49.4)	38.6 to 60.2
Conference Committee Member (n=71)	781	323 (41.4)	38.0 to 44.8

### Journals

In Turkish medical journals, women accounted for 27.2% of all editor-in-chief/editor positions, as presented in table 4. Women were significantly under-represented in the editor-in-chief position compared with their share of other editorial roles (p=0.007).

**Table 4:** Women’s representation in Turkish medical journal positions

Positions (n=journals)	Total number of positions	Number of women (%)	95% CIs
Editor-in-Chief/Editor (n=241)	361	98 (27.2)	22.8 to 32.0
Associate/Deputy/Co-/Managing/Section Editors (n=200)	2286	855 (37.4)	35.4 to 39.4
Editorial Board Members (n=237)	6225	2049 (32.9)	31.8 to 34.1

## Discussion

Women constituted two-fifths of Turkey’s medical-school workforce in 2023 (40.4%), yet held only 22.5% of deanships and 13.6% of surgical-division chairships, which is clear evidence of a leadership gap relative to their overall presence. This shortfall was quantified for each of 49 departments. Significant under-representation was found in only six departments, most notably paediatrics (36.1% chairs vs 57.9% faculty) and gynaecology-obstetrics (23.2% vs 41.6%), while the remaining 43 departments showed parity, for example, female leadership matched their faculty baseline. No department displayed a statistically significant excess of women in chair roles. At the school level, vice-deanships (38.5%) tracked the underlying female faculty share, indicating a position where no gap was detected.

In 2022, women comprised 34.2% of professors, 41.1% of associate professors and 48,6% of assistant professors in Turkish higher education.^11^ In medical schools, female academic staff accounted for 43.8%^12^ with women representing 36.1% of professors, 39.9% of associate professors and 44.6% of assistant professors,^11^ marking an increase from 2018.^13^ However, the rise in the number of faculty in Turkish medical schools^14^ contributing to increased women representation still lags behind the overall growth in female students and doctors, as observed in other countries^15^ and in higher education as a whole in Turkey.^16^ In terms of administrative positions, the percentage of female deans in Turkish medical schools was 14.0% in 2020.^11^ Although there has been an improvement over 3 years by reaching 22.5%, our findings point out a persistent gender gap in academic medicine leadership in Turkey.

Leadership in academic medicine extends beyond medical schools.^9^ In specialty boards and their conferences, women were not significantly under-represented as chairs once their share of all committee members was taken into account (boards 30.5% vs 36.5%; conferences 34.2% vs 41.1%). However, women held 27.2% of editor-in-chief roles in medical journals, significantly lower than their share of editorial board positions, indicating under-representation in top publishing leadership.

These findings show that, while a system-wide gender gap persists, driven chiefly by some disciplines and senior gate-keeping roles such as dean and editor-in-chief, many middle-tier or service positions already mirror the gender composition of their talent pool, underscoring where progress has been made and where targeted interventions are most needed. The findings also indicate entrenched gender biases and structural barriers.^17^ This aligns with global issues, where women’s representation in higher echelons of leadership lags despite increasing numbers of women in the medical profession. However, the similarity between the rates of women deans in Turkey (22.5%) and those in German-speaking countries, ranging from 15.5% to 23.5%,^18^ is noteworthy. Additionally, Turkey’s rate surpasses that of many other countries in the Global South, such as India, where the proportion of female physicians is below 20%,^19^ and Pakistan, where women go to medical school but fail to practice medicine,^19^ let alone the rates in leadership positions. As a Global South country, Turkey offers surprisingly favourable conditions for gender equity compared with others, potentially owing to its secular heritage stemming from Atatürk,^20^ who is the founder of the modern Turkish republic and successor of Ottoman reforms.

Certain departments demonstrated higher representation of women, with dermatology having the highest rate at 63.8%. Notably, some departments such as urology and orthopaedics had no women chairs. Across surgical departments, the percentage of women was low. These outcomes align with broader literature, where gender inequity research has predominantly focused on surgical areas, with orthopaedics and urology featuring prominently in such discussions^21 22^ and has identified various forms of discrimination against women.^23^

Considering that women were not significantly under-represented as chairs in medical conferences, Turkey’s position in this manner can be interpreted as good compared with Global North. For instance, a study of 98 conferences spanning 20 specialties held mostly in Global North found that men outnumbered women as invited lecturers, panellists and planning committee organisers by an approximate ratio of 2:1.^24^ Therefore, the rates in Turkey indicate near-optimal levels for providing equal opportunities to both genders in terms of medical conference leadership.

Lastly, journals serve as valuable indicators of educators’ active roles in their field and provide an important platform for women to represent themselves and their research. However, our research revealed that women held only 27.2% of all editor-in-chief positions, indicating a considerable gap. These findings align with the global evaluation of editorial board composition in academic medicine journals, which shows that 34.7% of the board members were women.^25^

Overall, our findings highlighted the gender equity issue in academic medicine in Turkey, raising concerns about potential problems. The significance of gender representation extends beyond women’s academic careers to impact healthcare,^26^ especially considering the presence of gender bias in medical diagnoses.^27^ This implicit bias could influence both medical and psychological diagnoses by being influenced by the patient’s gender. It also acts as a barrier to women’s academic progression, compounded by other factors such as the glass ceiling phenomenon—a subtle but persistent set of barriers hindering qualified women from accessing top management positions.^28^ Additionally, it is important that the victims of gender bias are not only women but also men, as a study showed that ‘men are evaluated more negatively in fields with a low representation of men, yet this cannot be explained by their skills’.^29^ Hence, our call for fairness extends to both genders. It is also important to remember that while ‘gender equity’ and ‘gender equality’ are often used interchangeably, achieving complete ‘equality’ between genders is neither possible nor the ultimate goal. The goal is not to treat all genders exactly the same but to ensure fairness and justice for everyone.^30^

Our study is limited by its cross-sectional design, which provides a snapshot but cannot establish causality. The data are also subject to the accuracy and completeness of publicly available information. We primarily extracted data from the websites of faculties, boards and conferences and journals. Data accuracy and completeness relied on publicly available sources, primarily websites of faculties, boards, conferences and journals, which were sometimes outdated or inaccessible. Data abstraction was performed by a single author per entity, posing a risk of bias or errors. Additionally, in this study, gender was considered as a binary construct for simplicity, and the method of gender recognition—relying on information from websites, photos or internet searches—may have led to misclassification or misrepresentation of some individuals due to the limitations of the available data.

We believe that acknowledging the problem is the initial step. Our research, exploring gender inequity in Turkish academic medicine comprehensively for the first time, indicates that there is considerable progress to be made. The next steps should involve analysing the causes, developing solutions and actively providing opportunities to both genders to pursue their aspirations in their careers. More advanced statistical methods and further research could explore the root causes of the gender disparities observed and identify effective strategies for promoting gender equity in medical leadership. Comparative studies between different countries and cultural contexts could also offer valuable perspectives on universal versus localised barriers and solutions. Future research should also evaluate the impact of specific interventions aimed at increasing women’s representation in medical leadership roles. Finally, although all study participants held comparable postgraduate medical qualifications, we could not adjust for upstream factors, such as gender differences in access to or length of tertiary education, that might shape the leadership pipeline; future individual-level research should explore these potential confounders.

## Conclusions

This study reveals gender disparities in leadership roles across Turkish academic medicine, particularly in senior positions. While many mid-level roles now reflect the gender makeup of the workforce, top positions, such as deans and editors-in-chief, show significant under-representation of women. These gaps, statistically validated against the available talent pool, point to structural barriers that require ongoing efforts. Strategic interventions should focus on addressing disparities in high-impact roles and supporting career progression.

## Data Availability

Data are available upon reasonable request.
