# Supplementary material for: Cross-sectional study of women’s representation in leadership positions in Turkish medical schools, academic departments, specialty boards, conferences and journals in 2023
Source: BMJ Open. 2025 Oct 13;15(10):e089298. doi: 10.1136/bmjopen-2024-089298 (PMC12519649; doi:10.1136/bmjopen-2024-089298)
Supplement: Supplementary file 1 [file bmjopen-2024-089298.reviewer_comments.pdf]

PEER REVIEW HISTORY

BMJ Open publishes all reviews undertaken for accepted manuscripts. Reviewers are asked to complete a checklist review form and are provided with free text boxes to elaborate on their assessment. These free text comments are reproduced below.

ARTICLE DETAILS

Title (Provisional)

A cross-sectional study of women's representation in leadership positions in Turkish medical schools, academic departments, specialty boards, conferences, and journals in 2023

Authors

Eroğlu, Fatma Sıla; Koyuncu, Sevil Buket; Erkan, Beyza; Kıyak, Yavuz Selim

---

VERSION 1 - REVIEW

**Reviewer**	**1**
**Name**	**Chatterjee, Archana**
**Affiliation**	**University of South Dakota**
**Date**	**18-Nov-2024**
**COI**	**None**

---

1. Why do you call it a Global South perspective when the study is only from Turkey?

2. Page 2, line 39 – change "women" to "women's"; line 51 – remove comma; line 52 – add period at end of sentence.

3. Page 3, line 11 – change "is" to "are"; line 18 – add "the" before "Turkish", change "women" to "women's"; line 30 – change "women" to "women's"; line 32 – change "it" to "they"; lines 44-47 - please use this corrected sentence – "Given that leadership positions in medical education are varied, there exists a significant research gap in comprehensively addressing women's representation in these positions, not only in the Turkish context but also on a global scale."

4. Page 4, line 7 – add "a" before "global"; delete "on Global South"; line 14 – change "process has been" to "was"

5. Page 5, line 14 – separate the two paragraphs; line 26 – change "women" to "women's"; line 27 – discuss "discuss" to "discussed"; line 29 – add "the" before "data"; line 39 – use this phrase plese – "of a shared understanding and the final version"; line 40 – change "was" to "were"; line 41 – add "via" before "website" and change "website" to "websites"; line 49 – change "In" to "For"; lines 50, 54 and 59 – change "women" to "women's"

6. Page 6, line 4 – change “in” to “from”; line 7, 19, 42 and 52– change “women” to “women’s”

7. Page 8, line 23 – add “were” before “held”; line 25 – change “women” to “women’s”, and use this phrase – “on specialty boards”; line 28, 30 and 36 – change “women” to “women’s”

8. Page 9, line 14 - change “women” to “women’s”; line 40 – add “faculty” after “women”;

9. Page 10, line 18 – add “In” before “Professional”; line 22 – change “for” to “than”; line 27 – change “women” to “women’s”; line 30 – add “the” before “Global”; line 42 – delete “from equality”

10. Page 11, line 5 – change “is” to “are”; line 39 – change “were” to “was”

11. Reference #s 22 and 27 are not cited in the text of the article. Please either delete these and renumber the references appropriately in the text or add them to the text.

12. Change in title - "Women" to "Women's"

**Reviewer**	**2**
**Name**	**Wong, Cynthia**
**Affiliation**	**University of Iowa Hospitals and Clinics, Anesthesiology**
**Date**	**29-Nov-2024**
**COI**	**None**

BMJ Open 2024_089298

General Comments: The manuscript is a cross-sectional study of women in academic medicine in Turkey in 2023. The authors summarize leadership in medical school faculty, specialty boards, conference planning leadership, and journal editorial boards. The information is important as it is well understood that women are underrepresented in academic medicine. Most reports have summarized the status of women in the Global North; data are lacking from the Global South. Baseline data are important for measuring progress.

Overall, the manuscript is comprehensive, but could be shortened for brevity and clarity of writing.

Major Comments:

1. The authors should emphasize the proportion of leadership who are women to the overall proportion of medical school faculty who are women. This determines whether women are underrepresented in leadership positions.

2. I encourage the authors to consider the difference between equality and equity in their discussion. Women and men may not ever be equal – there are real differences between the genders (e.g., only women give birth), but equity should be achievable.

3. P4L24: 15 of 128 medical schools (12%) were not included in the analysis because available information was not found on the Web. This is a high proportion. Did the authors make any attempt to obtain the data by other means?

4. How did the authors determine the job description/titles within each entity? Are these standardized? For example, does every medical school have a Vice Dean and a Faculty Board or Faculty Executive Committee? Does every professional society have a General Secretary and Treasurer?

5. The authors have not reported the amount of missing data. There appears to be a fair amount of missing data. For example, Table 1, only 102 deans are listed from 113 medical schools.

6. P5L44-P6, statistical analysis: It is unclear why the authors used the Mann Whitney U test to compare categorical data (women/men). Usually, the chi square of Fisher exact test is indicated.

7. Statistical analysis: In several circumstances, the authors appear to have made multiple comparisons of the same data. For example, Basic department data were compared to both Internal data and to Surgical data. Were P values corrected for these multiple comparisons?

8. P11L29-33: The abstract and manuscript conclusions should be supported by the data of the study. In the concluding paragraph, the authors state, "despite some progress." However, the authors did not assess progress. Neither did they formally compare their data to other Global South countries. These comparisons can be discussed in the Discussion section, but they should not be in the Conclusion paragraph.

Minor Comments:

1. Round all percent values in the manuscript to the nearest tenth.

2. The authors refer to the general category of medical school faculty as "medical education," but many faculty also perform research as their primary responsibility. Therefore, I think it more accurate to refer to the entire enterprise as "academic medicine" For example P3L35, I suggest, "However, leadership positions in academic medicine extend…" and P3L38, "In terms of academic medicine, the leadership positions…."

3. The language sometimes is lacking in scientific tone. For example, P4L3: use of "guiding light," P5L34: Use of "first leg" and "second leg."

4. The manuscript should be reviewed for minor errors in word usage and tense. For example, P4L13, use of "has been" (present perfect). Instead, the authors should use the past tense "was."

5. Report exact P values (P = xx), not P < or P >.

6. P9-11, Discussion section citations: The citations in the Discussion section are wrong – they appear to be off by one number (e.g., citation 14 should be 15, 15 should be 16, on so forth).

Specific Concerns:

1. Title: Add year of analysis to the title.

2. Abstract: The abstract does not appear to align with the template for the Journal's structured abstract.

3. P2L38-9: The authors did not compare Turkey to other countries; therefore, the abstract conclusion should not make this comparison. It can be discussed in the Discussion section of the manuscript (see Major Comment 9, above).

4. P2L50: Delete "minor."

5. P2L46: The Introduction is long. Some of this material can be deleted or moved to the Discussion section.

6. P3L5-8: Are these global data? Please specify.

7. P3L24: "Turkish medical schools" rather "facilities."

8. P3L28: Is this better stated, "…the rise in the number of faculty" rather than "doctors?"

9. P4L24-8: Please cite the source for the Council of Higher Education registry in Turkey.

10. P4L26: "Medical schools" rather than "medical facilities."

11. P4L30-8: When were these data collected?

12. P4L33: To my knowledge, the term "Internal Sciences" is not widely used or understood. Please define. Which departments were considered Basic, Surgical, and Internal for the purposes of this summary? Consider labeling the departments in Table 2 with their assignment to one of the three categories.

13. P4L42-5: Are "specialty boards" in Turkey the same as specialty professional societies? For example, in the United States, each specialty usually has a professional society (e.g., the American Society of Anesthesiologists) and a specialty board (e.g., the American Board of Anesthesiology). Are all of these organizations listed on the Turkish Medical Association website?

14. P5-6, statistical analysis: Report the software that was used for the statistical analysis. What P-value determined statistical significance?

15. P6L6-7: What was the rationale for comparing gender representation for journals indexed in the Web of Science and the Turkish index?

16. P6L13: I suggest reporting the overall percent of women faculty members first. Arguably, the proportion of leadership positions that are held by women should reflect this baseline proportion.

17. P9, Discussion: Throughout the Discussion section, there is much repetition of the results. Please limit this.

18. P9L41-2: The authors did not formally analyze the relationship between fewer women faculty and fewer women department chairs.

19. P9L43-45: If 22 department have 50% women, then one might expect (hope) that 50% of the chairs would be women (11 departments). 9/22 is not statistically different than 11/22.

20. P9L54-7: Why did the authors chose to compare their data with that of German-speaking countries specifically. There are data from many others countries.

21. P10L10: There is only 1 aerospace medicine department (Table 2). Thus, the finding of no women chairs hardly seems like a disparity.

22. P10L49-54: This is a run-on sentence and the meaning is not clear. Please clarify.

23. P11L3-10: A limitation is that gender is not a binary construct, although the authors have only assumed two genders.

24. P11L3-10: Another limitation is that only one author abstracted data for each entity. I stronger study design would have had 2 authors abstract data for each entity, then the data would be compared and discrepancies resolved.

Respectfully reviewed,

Cynthia A. Wong, MD

VERSION 1 - AUTHOR RESPONSE

Reviewer: 1

1. Why do you call it a Global South perspective when the study is only from Turkey?

***Response**: Thank you for this question. Since Turkey belongs to the Global South and offers "a" Global South perspective, we believed this title would effectively reflect the study's contribution.*

2. Page 2, line 39 – change "women" to "women's"; line 51 – remove comma; line 52 – add period at end of sentence.

***Response**: Done.*

3. Page 3, line 11 – change "is" to "are"; line 18 – add "the" before "Turkish", change "women" to "women's"; line 30 – change "women" to "women's"; line 32 – change "it"

to “they”; lines 44-47 - please use this corrected sentence – “Given that leadership positions in medical education are varied, there exists a significant research gap in comprehensively addressing women’s representation in these positions, not only in the Turkish context but also on a global scale.”

***Response**: Done.*

4. Page 4, line 7 – add “a” before “global”; delete “on Global South”; line 14 – change “process has been” to “was”

***Response**: Done.*

5. Page 5, line 14 – separate the two paragraphs; line 26 – change “women” to “women’s”; line 27 – discuss “discuss” to “discussed”; line 29 – add “the” before “data”; line 39 – use this phrase plese – “of a shared understanding and the final version”; line 40 – change “was” to “were”; line 41 – add “via” before “website” and change “website” to “websites”; line 49 – change “In” to “For”; lines 50, 54 and 59 – change “women” to “women’s”

***Response**: Done.*

6. Page 6, line 4 – change “in” to “from”; line 7, 19, 42 and 52– change “women” to “women’s”

***Response**: Done.*

7. Page 8, line 23 – add “were” before “held”; line 25 – change “women” to “women’s”, and use this phrase – “on specialty boards”; line 28, 30 and 36 – change “women” to “women’s”

***Response**: Done.*

8. Page 9, line 14 - change “women” to “women’s”; line 40 – add “faculty” after “women”;

***Response**: Done.*

9. Page 10, line 18 – add “In” before “Professional”; line 22 – change “for” to “than”; line 27 – change “women” to “women’s”; line 30 – add “the” before “Global”; line 42 – delete “from equality”

***Response**: Done.*

10. Page 11, line 5 – change “is” to “are”; line 39 – change “were” to “was”

***Response**: Done.*

11. Reference #s 22 and 27 are not cited in the text of the article. Please either delete these and renumber the references appropriately in the text or add them to the text.

***Response**: Thank you for addressing this important issue. We have carefully reviewed and corrected all citation-related errors.*

12. Change in title - "Women" to "Women's"

***Response**: Done.*

Reviewer: 2

General Comments: The manuscript is a cross-sectional study of women in academic medicine in Turkey in 2023. The authors summarize leadership in medical school faculty, specialty boards, conference planning leadership, and journal editorial boards. The information is important as it is well understood that women are underrepresented in academic medicine. Most reports have summarized the status of women in the Global North; data are lacking from the Global South. Baseline data is important for measuring progress. Overall, the manuscript is comprehensive, but could be shortened for brevity and clarity of writing.

***Response:** Thank you for your valuable feedback. We appreciate your recognition of the importance of our study.We’ve made some changes to improve clarity and brevity.*

Major Comments:

1. The authors should emphasize the proportion of leadership who are women to the overall proportion of medical school faculty who are women. This determines whether women are underrepresented in leadership positions.

***Response:** Thank you for your suggestion. We agree that this is significant data and have revised the manuscript accordingly: “Out of 49 departments, the percentage of women among faculty members exceeded the percentage of women in chair positions in 41 (83,7%) departments.”*

2. I encourage the authors to consider the difference between equality and equity in their discussion. Women and men may not ever be equal – there are real differences between the genders (e.g., only women give birth), but equity should be achievable.

Response: *Thank you for highlighting the important distinction between equality and equity. We agree that this distinction is essential in discussions about gender representation. In response, we have included a paragraph in the discussion section to explicitly address this distinction: "It is important to remember that while "gender equity" and "gender equality" are often used interchangeably, achieving complete "equality" between genders is neither possible nor the ultimate goal.The goal is not to treat all genders exactly the same but to ensure fairness and justice for everyone."*

3. P4L24: 15 of 128 medical schools (12%) were not included in the analysis because available information was not found on the Web. This is a high proportion. Did the authors make any attempt to obtain the data by other means?

Response*: Thank you for your observation. We agree that the exclusion of 12% of medical schools due to unavailable online information misses a high number of individuals. However, since our dataset is extensive and encompasses 88% of the total population, which allows us to generalize the findings, we decided not to adopt additional data collection methods.*

4. How did the authors determine the job description/titles within each entity? Are these standardized? For example, does every medical school have a Vice Dean and a Faculty Board or Faculty Executive Committee? Does every professional society have a General Secretary and Treasurer?

Response: *Thank you for highlighting this methodological aspect.The job descriptions and titles within each entity were determined based on commonly recognized administrative structures in medical schools and professional societies. While there may be some little variations across institutions, we aimed to use widely recognized titles. In medical schools, roles such as Vice Dean and Faculty Board/Faculty Executive Committee are present. Similarly, in professional societies, positions like General Secretary and Treasurer are commonly found, even if some organizations have additional roles. Therefore, we believe that our data collection process helped us to sustain a sufficient level of validity.*

5. The authors have not reported the amount of missing data. There appears to be a fair amount of missing data. For example, Table 1, only 102 deans are listed from *113 medical schools.*

Response: *Thank you for your observation. We acknowledge that some data on positions in medical schools, specialty boards, conferences, and journals were missing due to unavailable*

online information, which we stated as a limitation of our study. Given the large scope of our dataset, we could not list each instance of missing data in the manuscript due to the extensive number of positions analyzed and space limitations. However, the proportion of missing data does not prevent generalization as it represents a relatively small fraction of the overall dataset.

6. P5L44-P6, statistical analysis: It is unclear why the authors used the Mann Whitney U test to compare categorical data (women/men). Usually, the chi square of Fisher exact test is indicated.

Response: *Thank you for your comment. We used the Mann-Whitney U test because our analysis involved comparing the distribution of women's representation (as percentages) across different department categories rather than individual categorical counts.*

7. Statistical analysis: In several circumstances, the authors appear to have made multiple comparisons of the same data. For example, Basic department data were compared to both Internal data and to Surgical data. Were P values corrected for these multiple comparisons?

Response: *Thank you for your comment. Due to financial limitations, we could not apply further statistics such as multiple comparisons correction in our analysis. Our primary, and also obligatory, target was to explore potential differences between groups rather than conduct a formal hypothesis-driven multiple testing correction. We added this as a limitation that can be addressed in further research: "More advanced statistical methods and further research could explore the root causes of the gender disparities observed ..."*

8. P11L29-33: The abstract and manuscript conclusions should be supported by the data of the study. In the concluding paragraph, the authors state, "despite some progress." However, the authors did not assess progress. Neither did they formally compare their data to other Global South countries. These comparisons can be discussed in the Discussion section, but they should not be in the Conclusion paragraph.

Response: *The conclusions in both the abstract and the main text were updated accordingly.*

Minor Comments:

1. Round all percent values in the manuscript to the nearest tenth.

Response*: Done.*

2. The authors refer to the general category of medical school faculty as "medical

education," but many faculty also perform research as their primary responsibility. Therefore, I think it more accurate to refer to the entire enterprise as "academic medicine" For example P3L35, I suggest, "However, leadership positions in academic medicine extend..." and P3L38, "In terms of academic medicine, the leadership positions...."

***Response**: Thank you for this suggestion. We believe 'medical education' is the appropriate term, as it covers all three phases of medical education (undergraduate (e.g. medical schools), postgraduate (e.g. medical schools), and continuous (e.g. specialty boards, journals, conferences)). We covered all three stages of medical education.*

3. The language sometimes is lacking in scientific tone. For example, P4L3: use of "guiding light," P5L34: Use of "first leg" and "second leg."

***Response**: Thank you for your comment. We have thoroughly reviewed the manuscript and made the necessary revisions to ensure a more consistent scientific tone.*

4. The manuscript should be reviewed for minor errors in word usage and tense. For example, P4L13, use of "has been" (present perfect). Instead, the authors should use the past tense "was."

***Response**: Thank you for your feedback. We have examined the manuscript for grammar and spelling errors and have made the necessary corrections.*

5. Report exact P values (P = xx), not P < or P >.

***Response:** Since several comparisons were conducted, reporting each exact P-value individually was not feasible. Therefore, we grouped the results and reported them in categories of P < 0.05 and P < 0.01 for clarity and conciseness.*

6. P9-11, Discussion section citations: The citations in the Discussion section are wrong – they appear to be off by one number (e.g., citation 14 should be 15, 15 should be 16, on so forth).

***Response**: Thank you for addressing this important issue. We have carefully reviewed and corrected all citation-related errors.*

Specific Concerns:

1. Title: Add year of analysis to the title.

***Response**: Thank you for your suggestion. We agree that this addition enhances the clarity and relevance of the title. The title has been updated: "Women's Representation in Leadership*

Positions of Medical Schools and Departments, Specialty Boards and Conferences, and Journals in Turkey in 2023: Cross-Sectional Data from 17939 Faculty Members, 112 Boards, 73 Conferences, and 246 Journals"

2. Abstract: The abstract does not appear to align with the template for the Journal's structured abstract.

Response: *Thank you for your feedback. We have carefully reviewed the abstract and made the necessary adjustments to ensure it aligns with the journal's structured abstract template. The revised version is included in the uploaded document.*

3. P2L38-9: The authors did not compare Turkey to other countries; therefore, the abstract conclusion should not make this comparison. It can be discussed in the Discussion section of the manuscript (see Major Comment 9, above).

Response: *The conclusion in the abstract were updated accordingly. (P.S. We could not find Major Comment 9 above)*

4. P2L50: Delete "minor."

Response*: Done.*

5. P2L46: The Introduction is long. Some of this material can be deleted or moved to the Discussion section.

6. P3L5-8: Are these global data? Please specify.

Response: *Thank you for bringing this to our attention. Yes, the data mentioned are global. We have revised the text for clarity: "As highlighted in the United Nations Sustainable Development Goals Report 2023, despite progress, women globally held 28.2 percent of management positions in 2021, with over 140 years projected to achieve gender parity in these positions"*

7. P3L24: "Turkish medical schools" rather "facilities."

Response*: Done.*

8. P3L28: Is this better stated, "...the rise in the number of faculty" rather than "doctors?"

Response*: Thank you for your suggestion. We initially used the term "doctors" to align with the phrasing in the reference we cited. However, we understand that "faculty" may better convey*

the context of our study. To enhance clarity and precision, we have revised the wording to "faculty" in the relevant section of the manuscript.

9. P4Ls."24-8: Please cite the source for the Council of Higher Education registry in Turkey.

***Response**: Done.*

10. P4L26: "Medical schools" rather than "medical facilities".

***Response**: Done.*

11. P4L30-8: When were these data collected?

***Response:** Thank you for your question. As stated in the Methods section, "The data collection process was conducted between August and December 2023."*

12. P4L33: To my knowledge, the term "Internal Sciences" is not widely used or understood. Please define. Which departments were considered Basic, Surgical, and Internal for the purposes of this summary? Consider labeling the departments in Table 2 with their assignment to one of the three categories.

***Response:** Thank you for pointing this out. We acknowledge that "Internal Sciences" may not be a widely recognized term. In our classification, we used "Internal Sciences" to refer to clinical, non-surgical disciplines, typically encompassing specialties such as Internal Medicine, Pediatrics, Neurology, and similar fields. "Surgical Sciences" refers to any specialty that involves surgical procedures, such as General Surgery, Ophthalmology, or related fields like Pathology that support surgical work. "Basic Sciences" refers to non-clinical specialties, which include disciplines like Anatomy, Physiology, Biochemistry, and others that do not directly involve patient care. We added this explanation to the methods section.*

For clarity, we categorized departments in Table 2 into three groups: Basic, Surgical, and Internal Sciences and explained the terminology at the beginning of the Results, paragraph 2.

13. P4L42-5: Are "specialty boards" in Turkey the same as specialty professional societies? For example, in the United States, each specialty usually has a professional society (e.g., the American Society of Anesthesiologists) and a specialty board (e.g., the American Board of Anesthesiology). Are all of these organizations listed on the Turkish Medical Association website?

Response: Thank you for your insightful question. In our study, we classified specialty boards as equivalent to specialty professional societies, differing from the distinction made in the

United States. In Turkey, generally these organizations serve both certifying and professional functions within their respective specialties. Additionally, as noted, all of these organizations are officially listed on the Turkish Medical Association website.

14. P5-6, statistical analysis: Report the software that was used for the statistical analysis. What P-value determined statistical significance?

Response: *Thank you for pointing out an important aspect we overlooked. We added: "Jamovi (version: 2.2.5), an R-based open-source software, was used for the statistical analysis. A P-value threshold of 0.05 was used to determine statistical significance."*

15. P6L6-7: What was the rationale for comparing gender representation for journals indexed in the Web of Science and the Turkish index?

Response: *Thank you for your question. The rationale for comparing gender representation between journals indexed in the Web of Science and the Turkish index (TR Dizin) was to assess whether differences exist in the gender representation of editorial boards at the national and international levels. Web of Science-indexed journals include publications from across the globe, providing a broader, international perspective on gender representation. In contrast, TR Dizin-indexed journals primarily focus on Turkey, reflecting the country's academic and scientific context, since one of TR Dizin's main goals is to create resources that represent Turkey's scientific knowledge. To clarify this distinction, we have revised the manuscript as follows: "To identify medical journals in Turkey, we conducted a search using Web of Science and Turkish index (TRDizin), a national database, to compare journals at national and international levels.. "*

16. P6L13: I suggest reporting the overall percent of women faculty members first. Arguably, the proportion of leadership positions that are held by women should reflect this baseline proportion.

Response: *Thank you for the suggestion. We understand the importance of comparing the proportion of women in leadership positions with the overall representation of women faculty members. However, the data we used was sourced globally and included many professions other than medicine, and unfortunately, it does not provide the baseline information on the overall proportion of women faculty members.*

In fact, this lack of data on gender distribution in faculty positions is one of the key reasons we initiated this research. We aim to fill this gap and provide a clearer understanding of gender representation in academic leadership across different institutions.

17. P9, Discussion: Throughout the Discussion section, there is much repetition of the results. Please limit this.

Response: *Thank you for your feedback. We have revised the Discussion section to reduce repetition of the results and ensure that it focuses more on relevant findings in relation to existing literature and references. The updated version now emphasizes comparisons with previous studies and avoids restating the results section. We appreciate your suggestion and believe these changes improve the clarity and conciseness of the discussion.*

18. P9L41-2: The authors did not formally analyze the relationship between fewer women faculty and fewer women department chairs.

Response: *Thank you for your comment. We appreciate the suggestion to formally analyze the relationship between fewer women faculty and fewer women department chairs. We have incorporated this into our manuscript as follows: "Out of 49 departments, the percentage of women among faculty members exceeded the percentage of women in chair positions in 41 (83,7%) departments."*

19. P9L43-45: If 22 departments have 50% women, then one might expect (hope) that 50% of the chairs would be women (11 departments). 9/22 is not statistically different than 11/22.

Response: *Thank you for your insightful comment. We see how the phrasing might have caused confusion. To clarify, when we stated that "among the 22 departments where women held at least 50% of the positions, only 9 of them had at least 50% of the women chairs," we were referring to the proportion of women within each department, specifically the leadership positions in those departments.*

In other words, while the overall positions in those departments are 50% women, the leadership within those departments does not always reflect this proportion.

We adjusted the wording in the manuscript, including our adjustion on revision number 18: "This may suggest that fewer female chairs explained by a fewer women population in the department, however, among the 22 departments where women held at least 50% of the positions, only 9 of those 22 departments had at least 50% of the women chairs."

20. P9L54-7: Why did the authors chose to compare their data with that of German-speaking countries specifically. There are data from many others countries.

Response: *Thank you for your question. The comparison with German-speaking countries was chosen because the referenced study was conducted recently and includes multinational findings. These aspects make it a relevant and up-to-date instance for comparison. Additionally, Europe's geographical proximity to Turkey and Turkey's efforts to enter EU makes it a meaningful point of reference. Furthermore, our study does not solely compare Turkey to German-speaking countries; we also included comparisons with other Global South countries.*

21. P10L10: There is only 1 aerospace medicine department (Table 2). Thus, the finding of no women chairs hardly seems like a disparity.

Response*: Thank you for this observation. You are correct that with only one aerospace medicine department, the absence of a woman chair does not represent a meaningful disparity due to the small sample size. We have clarified this in the manuscript: "However, aerospace medicine has only one chair position in total, so this is hardly indicative of systemic inequality."*

22. P10L49-54: This is a run-on sentence and the meaning is not clear. Please clarify.

Response*: Done.*

23. P11L3-10: A limitation is that gender is not a binary construct, although the authors have only assumed two genders.

Response: *Thank you for this valuable observation. We acknowledge that gender is not a binary construct; however, our study operates under the assumption of two genders due to the constraints of the available data sources. The gender information was derived from publicly accessible names and photos, which primarily classify individuals into binary categories. We recognize this as a limitation of our study and have addressed it in the manuscript as follows: "Additionally, in this study, gender was considered as a binary construct for simplicity, and the method of gender recognition—relying on information from websites, photos, or internet searches—may have led to misclassification or misrepresentation of some individuals due to the limitations of the available data."*

24. P11L3-10: Another limitation is that only one author abstracted data for each entity. A stronger study design would have had 2 authors abstract data for each entity, then the data would be compared and discrepancies resolved.

Response*: Thank you for highlighting this limitation. We agree that involving two authors in the data abstraction process would have strengthened the study design and enhanced the reliability of the findings.However, due to time constraints, as well as the large volume of data, a single author performed data abstraction for each entity. This limitation has been addressed in the manuscript as follows: "Data abstraction was performed by a single author per entity, posing a risk of bias or errors."*

VERSION 2 - REVIEW

**Reviewer**	**2**
**Name**	**Wong, Cynthia**
**Affiliation**	**University of Iowa Hospitals and Clinics, Anesthesiology**
**Date**	**05-Apr-2025**
**COI**	

BMJ Open 2024_089298R1

General Comments: The manuscript is a revision of a cross-sectional study of women in academic medicine in Turkey in 2023. I thank the authors for their detailed response to my previous questions. However, I continue to have some major concerns about the study analysis, result reporting, and conclusions not supported by data. The authors have not addressed many of my initial comments/questions/suggestions.

Major Comments:

1. P10L15-19, P12L8-12, P2L39-41: The authors have not presented any data to support "significant underrepresentation." To do this, they must compare the percent women deans (and other leadership positions) to a baseline, for example, the proportion of all women physicians, all women faculty members, or all women in society.

2. In my opinion, the authors have not adequately addressed the missing data – essentially, they are just ignoring it. Given that the data set is incomplete, the authors should report the 95% confidence intervals of all their outcomes. The amount of missing data should be reported in the Results section.

3. The statistical analysis should be reviewed by a biostatistician. I am not a statistician, but I do not believe the analyses are valid. For example, categorical data (proportion of women/men) should be compared using an appropriate test (e.g., Chi squared, not Mann-Whitney U). The authors have made multiple comparisons without correcting P-values. Finally, that have not report exact P-values as requested, but rather "<" or ">." For example, P6L55-58, P9L13, why are the exact P-values not reported?

4. As noted in my previous review, "equality" is not achievable nor desirable, but equity is. The authors have continued to use the work "equality" throughout the manuscript rather than the suggested word, "equity."

Minor Comments:

1. While I agree with the authors that medical education includes all phases of medical education (undergraduate, graduate, and postgraduate), I continue to disagree that "medical education" is the appropriate term for this study. The dean of a medical school leads the

entire enterprise, which traditionally, has several missions, including education and research. The term “medical education” does not encompass editorial boards of medical journals (P3L32, P4L8). Thus, I believe the term “academic medicine” is more appropriate. Please change all “in medical education” to “in academic medicine” throughout the manuscript.

Specific Concerns:

1. I suggest simplifying the title: “A cross-sectional study of women’s representation in leadership positions in Turkish medical schools, academic departments, specialty boards, conferences, and journals in 2023.”

2. P2L35: Please specify “Turkish medical journals.”

3. P2L39: Fewer than what? The comparator must be listed.

4. P2L45-9: Missing data is another limitation.

5. P3-4, Introduction: As noted previously, the Introduction should be shortened. If the authors wish to retain some of this information, it can be moved to the Discussion section.

6. P3L28-30: From what year are these dean data?

7. P5L18-32: How were women identified? What assumptions were made?

8. Table 2: I suggest switching columns 1 and 2. List the departments in column 1 and the classification in column 2. Alternatively, eliminate the classification column and divide the table into 3 sections: internal, surgical, basic, and list the departments in order underneath. In the table, how was the department order determined? I suggest alphabetical.

9. P10L21-22: Again, the authors did not do this statistical analysis, therefore, they cannot draw this conclusion (noted on previous review).

1. P10L22-6: P9L43-45: If 22 department have 50% women, then one might expect (hope) that 50% of the chairs would be women (11 departments). 9/22 is not statistically different than 11/22 (noted on the previous review).

Respectfully reviewed,

Cynthia A. Wong, MD

**Reviewer**	**3**
**Name**	**Moinuddin, Mohammed**
**Affiliation**	**University of Lancashire, School of Medicine and Dentistry**
**Date**	**20-May-2025**
**COI**	**None**

Gender equity in the leadership roles of Turkish medical schools, speciality boards, conferences, and medical journals in 2023 was explored. An interesting research topic, however, the statistical analysis needs further consideration and clarification. My specific observations are below:

Comment 1 – "Mann-Whitney U test was carried out to compare women's representation in basic (n=12), internal (n=23), and surgical (n=14) departments." It is surprising how the Mann-Whitney U test was applied. This is used to compare the distribution of continuous variables when data do not follow a normal distribution. From Table 1, I can see that the hypothesis testing problem can be seen as testing the association between gender and types of positions. The statistical significance of this association can be assessed using a Chi-square test or a similar type of test. Another issue is that the data looks clustered because there are multiple members from the same school. Therefore, just applying a simple Chi-square will not be enough. Same comments for Tables 3 and 4.

Comment 2 – The authors used hypothesis testing to see if there is a difference in the proportion of females in the position. It could be interesting to see some measure of association, for example odds ratio.

Comment 3 – I was wondering how the authors handled the missing values. Please clarify. In addition, I do not see any discussion of any confounders. A strong confounder could be the educational level of the participants and the educational level in the country by gender. If overall, the educational attainment is higher among men compared to women, especially at the tertiary level, then that might explain the inequality. Without addressing these issues, it is difficult to assess the validity of the conclusion.

VERSION 2 - AUTHOR RESPONSE

Reviewer: 2

General Comments:

- The manuscript is a revision of a cross-sectional study of women in academic medicine in Turkey in 2023. I thank the authors for their detailed response to my previous questions. However, I continue to have some major concerns about the study analysis, result reporting, and conclusions not supported by data. The authors have not addressed many of my initial comments/questions/suggestions.
    - ***Response:*** *Thank you for your valuable comments. We acknowledge the validity of your concerns, especially regarding the appropriateness of statistical tests and the strength of the conclusions drawn in our initial submission. Now we recognized shortcomings in our earlier analysis and reporting.*

As a result, we chose the correct test by replacing inappropriate analyses (e.g., Mann-Whitney U for categorical data) with more appropriate tests, including Pearson's χ^2 test with continuity correction for categorical comparisons and added 95% confidence intervals throughout. These revisions revealed important changes in findings such as the absence of statistically significant gender disparities in some areas previously thought inequitable.

Accordingly, we revised the manuscript to reflect these updated results and removed or rephrased conclusions that were previously unsupported. This process substantially improved the study, and we are grateful for your role in this revision. We hope the revised manuscript now addresses your previous concerns more comprehensively.

Major Comments:

- P10L15-19, P12L8-12, P2L39-41: The authors have not presented any data to support "significant underrepresentation." To do this, they must compare the percent women deans (and other leadership positions) to a baseline, for example, the proportion of all women physicians, all women faculty members, or all women in society.
    - ***Response:*** *We conducted the analysis with the correct type of tests and updated the article based on the new findings.*
- In my opinion, the authors have not adequately addressed the missing data – essentially, they are just ignoring it. Given that the data set is incomplete, the authors should report the 95% confidence intervals of all their outcomes. The amount of missing data should be reported in the Results section.
    - ***Response:*** *We added an explanation regarding how we handled missing data (to the methods section): "We faced two tiers of missing data across all four data sets (medical schools, specialty boards, conferences, journals). First, a small group of institutions (e.g., 15 out of 128 medical schools) had no accessible data even after our efforts to find, so they were excluded list-wise. Second, in the collected data, blank gender cells were uncommon (<5%) and were handled by excluding them from the variable-specific denominators when calculating proportions. We reported the number of observations available for each leadership position (e.g., Deans = 102/113) was provided, and 95% confidence intervals were calculated."*

      We also reported confidence intervals for all of the outcomes. See the results section. However, we could not find a feasible way to add the amount of missing data for each specific instance. We reported that the data was collected from how many medical schools in the first column of the tables with "n= ...". For example, Table 1, although there were 113 medical schools in total, we were able to collect data from 102 medical schools for deans, 97 for vice deans, 96 for faculty board members, so on. Therefore, we believe that we transparently report the amount of missing data: e.g. 113-102=11 for dean data. We also stated the missing data as a limitation.

- The statistical analysis should be reviewed by a biostatistician. I am not a statistician, but I do not believe the analyses are valid. For example, categorical data (proportion of women/men) should be compared using an appropriate test (e.g., Chi squared, not Mann-Whitney U). The authors have made multiple comparisons without correcting P-values. Finally, that have not report exact P-values as requested, but rather “<” or “>.” For example, P6L55-58, P9L13, why are the exact P-values not reported?
    - ***Response:*** *All leadership-vs-baseline comparisons that involve counts of women/men are now analysed with Pearson’s* χ^2 *test with continuity correction. For each one, we constructed a 2 x 2 table (women vs men × position vs others) and tested the null hypothesis that the proportion of women is the same in the role as in the all. Every test result in the tables and text is now presented with its exact P-value (to three significant digits) and a 95% confidence interval for the proportion, rather than the previous ‘<’ or ‘>’ notation. Example: Table 1 now reports* $p < 0.001$*, 0.62, 0.003, etc.*

- As noted in my previous review, “equality” is not achievable nor desirable, but equity is. The authors have continued to use the work “equality” throughout the manuscript rather than the suggested word, “equity.”
    - ***Response:*** *Thank you for your suggestion. The term “equality” has been replaced with “equity” throughout the manuscript.*

Minor Comments:

- While I agree with the authors that medical education includes all phases of medical education (undergraduate, graduate, and postgraduate), I continue to disagree that “medical education” is the appropriate term for this study. The dean of a medical school leads the entire enterprise, which traditionally, has several missions, including education and research. The term “medical education” does not encompass editorial boards of medical journals (P3L32, P4L8). Thus, I believe the term “academic medicine” is more appropriate. Please change all “in medical education” to “in academic medicine” throughout the manuscript.
    - ***Response:*** *Thank you for your suggestion. The term “medical education” has been replaced with “academic medicine” throughout the manuscript.*

Specific Concerns:

- I suggest simplifying the title: “A cross-sectional study of women’s representation in leadership positions in Turkish medical schools, academic departments, specialty boards, conferences, and journals in 2023.”
    - ***Response:*** *Thank you for your suggestion. We have simplified the title.*

- P2L35: Please specify “Turkish medical journals.”
    - ***Response:*** *Done.*

- P2L39: Fewer than what? The comparator must be listed.
    - ***Response:*** *We updated the abstract and solved this issue.*

- P2L45-9: Missing data is another limitation.

    - ***Response:*** *Thank you for your suggestion. We have updated the manuscripts as follows: "Potential inaccuracies due to missing or outdated information in publicly available data sources."*

- P3-4, Introduction: As noted previously, the Introduction should be shortened. If the authors wish to retain some of this information, it can be moved to the Discussion section.
    - ***Response:*** *We shortened the introduction by moving some information to the discussion.*

- P3L28-30: From what year are these dean data?
    - ***Response:*** *From 2020. Added.*

- P5L18-32: How were women identified? What assumptions were made?
    - ***Response:*** *We identified via inference from typical Turkish given names and visual inspection of photographs: "... data were collected through the official websites, and gender recognition was based on information available on websites, photos, or internet searches (inference from typical Turkish given names and visual inspection of photographs)."*

- Table 2: I suggest switching columns 1 and 2. List the departments in column 1 and the classification in column 2. Alternatively, eliminate the classification column and divide the table into 3 sections: internal, surgical, basic, and list the departments in order underneath. In the table, how was the department order determined? I suggest alphabetical.
    - ***Response:*** *Thank you for this suggestion. The table has been reorganized into three sections, the classification column has been removed, and departments are now listed alphabetically within each relevant section.*

- P10L21-22: Again, the authors did not do this statistical analysis, therefore, they cannot draw this conclusion (noted on previous review).
    - ***Response:*** *We conducted the analysis with the correct type of tests and updated the discussion based on the new findings.*

- P10L22-6: P9L43-45: If 22 department have 50% women, then one might expect (hope) that 50% of the chairs would be women (11 departments). 9/22 is not statistically different than 11/22 (noted on the previous review).
    - ***Response:*** *We conducted the analysis with the correct type of tests and updated the discussion based on the new findings.*

Reviewer: 3

Specific Concerns:

- "Mann-Whitney U test was carried out to compare women's representation in basic (n=12), internal (n=23), and surgical (n=14) departments." It is surprising how the Mann-Whitney U test was applied. This is used to compare the distribution of continuous variables when data do not follow a normal distribution. From Table 1, I can see that the hypothesis testing problem can be seen as testing the association between gender and types of positions. The statistical significance of this association can be assessed using a Chi-square test or a similar type of test. Another issue is that the data looks clustered because there are multiple members from the same school. Therefore, just applying a simple Chi-square will not be enough. Same comments for Tables 3 and 4.
    - ***Response:*** *As you recommended, we removed Mann-Whitney U test and used Chi-square test to assess the associations.*
- The authors used hypothesis testing to see if there is a difference in the proportion of females in the position. It could be interesting to see some measure of association, for example odds ratio.
    - ***Response:*** *This is a student-led project without financial support. Therefore, unfortunately, we could not receive support for conducting some further tests that might be interesting to see.*
- I was wondering how the authors handled the missing values. Please clarify. In addition, I do not see any discussion of any confounders. A strong confounder could be the educational level of the participants and the educational level in the country by gender. If overall, the educational attainment is higher among men compared to women, especially at the tertiary level, then that might explain the inequality. Without addressing these issues, it is difficult to assess the validity of the conclusion.
    - ***Response:*** *We added an explanation regarding how we handled missing data (to the methods section: We added an explanation regarding how we handled missing data (to the methods section): "Information that could not be located on an institution's public website was coded as missing (blank cell) at the time of extraction. Across the entire data set this affected ≤ 12 % of medical-school leadership cells, ≤ 5 % of specialty-board and conference cells, and < 3 % of journal-editorial cells. All denominators in the results tables therefore reflect the number of non-missing observations, and 95% confidence intervals were calculated to account for the reduced sample size where relevant."*

      We also reported confidence intervals for all of the outcomes. See the results section. However, we could not find a feasible way to add the amount of missing data for each specific instance. We reported that the data was collected from how many medical schools in the first column of the tables with "n= …". For example, Table 1, although there were 113 medical schools in total, we were able to collect data from 102 medical schools for deans, 97 for vice deans, 96 for faculty board members, so on. Therefore, we believe that we transparently report the amount of missing data: e.g. 113-102=11 for dean data.

      Regarding the confounders, all individuals included in our dataset had already completed both a medical degree and the specialty training required for faculty,

board, conference-committee or journal-editor roles, which largely equalises formal educational attainment within the study sample. Nonetheless, upstream disparities in access to tertiary education could influence the leadership pipeline and therefore merit acknowledgement.

We have therefore added the following sentence to the Limitations paragraph of the discussion: "Finally, although all study participants held comparable postgraduate medical qualifications, we could not adjust for upstream factors, such as gender differences in access to or length of tertiary education, that might shape the leadership pipeline; future individual-level research should explore these potential confounders."

VERSION 3 - REVIEW

**Reviewer**	**3**
**Name**	**Moinuddin, Mohammed**
**Affiliation**	**University of Lancashire, School of Medicine and Dentistry**
**Date**	**13-Aug-2025**
**COI**	

I thank the authors for their further correction and explanation.

- Please make sure that the explanation about the missing value handling is in the method section, which I could not find in the current version.

- I can see the section about confounders is properly inserted in the method section. I appreciate it.

VERSION 3 - AUTHOR RESPONSE

Reviewer: 3

Comments to the Author:
I thank the authors for their further correction and explanation.

- Please make sure that the explanation about the missing value handling is in the method section, which I could not find in the current version.
Response: Thank you. We have updated the methods section to include a clear explanation, as per your and the editor's request.

- I can see the section about confounders is properly inserted in the method section. I appreciate it.
Response: Thank you for your feedback. We appreciate your review.
